# Modulating disease-relevant tau oligomeric strains by small molecules

**DOI:** 10.1074/jbc.RA120.014630

**Published:** 2020-07-31

**Authors:** Filippa Lo Cascio, Stephanie Garcia, Mauro Montalbano, Nicha Puangmalai, Salome McAllen, Andrea Pace, Antonio Palumbo Piccionello, Rakez Kayed

**Affiliations:** 1 Mitchell Center for Neurodegenerative Diseases, University of Texas Medical Branch, Galveston, Texas, USA; 2 Departments of Neurology, Neuroscience, and Cell Biology, University of Texas Medical Branch, Galveston, Texas, USA; 3 Department of Biological, Chemical, and Pharmaceutical Sciences and Technologies-STEBICEF, University of Palermo, Palermo, Italy

**Keywords:** brain-derived tau oligomers, tau aggregation, tau oligomeric strains, toxicity, tau protein, tauopathy, small molecule, protein aggregation, oligomer

## Abstract

The pathological aggregation of tau plays an important role in Alzheimer's disease and many other related neurodegenerative diseases, collectively referred to as tauopathies. Recent evidence has demonstrated that tau oligomers, small and soluble prefibrillar aggregates, are highly toxic due to their strong ability to seed tau misfolding and propagate the pathology seen across different neurodegenerative diseases. We previously showed that novel curcumin derivatives affect preformed tau oligomer aggregation pathways by promoting the formation of more aggregated and nontoxic tau aggregates. To further investigate their therapeutic potential, we have extended our studies o disease-relevant brain-derived tau oligomers (BDTOs). Herein, using well-characterized BDTOs, isolated from brain tissues of different tauopathies, including Alzheimer's disease, progressive supranuclear palsy, and dementia with Lewy bodies, we found that curcumin derivatives modulate the aggregation state of BDTOs by reshaping them and rescue neurons from BDTO-associated toxicity. Interestingly, compound CL3 showed an effect on the aggregation pattern of BDTOs from different tauopathies, resulting in the formation of less neurotoxic larger tau aggregates with decreased hydrophobicity and seeding propensity. Our results lay the groundwork for potential investigations of the efficacy and beneficial effects of CL3 and other promising compounds for the treatment of tauopathies. Furthermore, CL3 may aid in the development of tau imaging agent for the detection of tau oligomeric strains and differential diagnosis of the tauopathies, thus enabling earlier interventions.

Alzheimer's disease (AD) is the most common form of dementia and the most prevalent progressive neurodegenerative disease associated with age. The major neuropathological features of AD are synaptic and neuronal degeneration and the presence of amyloid plaques and neurofibrillary tangles (NFTs), comprised of the intracellular inclusion of hyperphosphorylated tau protein (1, 2). Increasing evidence has revealed that NFTs in AD brains describe the progression of AD pathology more accurately than amyloid deposition, and post-mortem brain histopathology can be used to stage AD (3, 4). Hence, these observations suggest that tau aggregation plays a crucial role in mediating neurodegeneration and cognitive decline in AD and related diseases. Indeed, NFTs are not exclusive inclusions of AD, as these lesions are also characteristic of other pathologies, collectively referred to as tauopathies, including progressive supranuclear palsy (PSP), dementia with Lewy bodies (DLB), Pick's disease, frontotemporal dementia, and several others (5–9). Therefore, understanding the pathological function and role of tau is a challenge to identify new therapeutic agents and approaches (10, 11).

In recent years, the concept of “prion-like” induction and spreading of pathogenic proteins has been proposed for many neurodegenerative diseases (12–14). Indeed, researchers have started to consider tau as well as other amyloid proteins, including fibrils of β-amyloid (15, 16) and α-synuclein (17, 18), as prion-like due to their characteristic ability to template the misfolding and aggregation of native proteins, leading to the formation of distinct conformations known as “strains.” It has been shown that within the same aggregation state, tau exhibits conformational differences that could exert diverse downstream effects (19, 20). To date, the vast majority of the tau strain studies have been carried out on fibrillar tau aggregates (19–22); however, recent evidence has demonstrated that the small and soluble prefibrillar aggregates, tau oligomers (TauO), are highly toxic due to their strong ability to seed tau misfolding and propagate the pathology seen across different neurodegenerative diseases (23–25).

Despite the extensive research aimed at finding approaches to halt the progression of AD and related diseases, to date, there are no effective therapeutics able to stop or reverse the disease process and cognitive decline (26, 27). Therefore, early diagnosis of these disorders, prior to the progression of the pathology, may enhance the chances of success of potential therapeutic approaches (28–31). Over the past 10 years, many approaches targeting tau aggregates, including small molecules, have been an area of great interest for drug discovery to develop effective disease-modifying therapeutics (11, 32–35).

Curcumin is a plant-derived polyphenol that has been proposed as a promising candidate for the development of preventive and therapeutic approaches for many diseases, including neurodegenerative disorders (36–40). However, curcumin therapeutic application for AD and other neurodegenerative diseases is challenging due to its poor water solubility and low oral bioavailability (41, 42). Consequently, many alternative formulations and drug delivery systems, including nano-based approaches, have been developed in an effort to boost and improve its systemic bioavailability (43–47).

Recently, we showed that newly synthesized curcumin derivatives/analogs, with low toxicity profile, can interact with preformed toxic tau oligomers by reshaping their conformation, resulting in the formation of larger tau structures with decreased toxicity in human neuroblastoma cells and primary cortical neurons (48). In the current study, we expanded this investigation to disease-relevant tau oligomers isolated from AD, DLB, and PSP brain tissues. These brain-derived tau oligomers (BDTOs), alone or pretreated with novel curcumin derivatives, were thoroughly characterized and investigated biochemically and biophysically to assess their unique and distinct aggregation patterns and structures. Furthermore, we evaluated the effects of these compounds on BDTO uptake by cells and associated toxicity and their seeding potency in primary cortical neurons and FRET-biosensor cells.

## Results

### Characterization of disease-relevant tau oligomers

We have previously established the isolation of disease-relevant BDTOs to directly test whether tau oligomers form polymorphs or conformationally distinct strains that depend upon disease differences (23, 49–51). To characterize disease-relevant tau oligomeric strains, BDTOs were isolated from AD, DLB, and PSP brain tissues by immunoprecipitation using the tau oligomer–specific antibody, T22. The BDTOs were amplified using full-length Tau-441 (2N4R) recombinant monomer (TauM) and thoroughly characterized (23, 50, 51).

**Figure 1.**
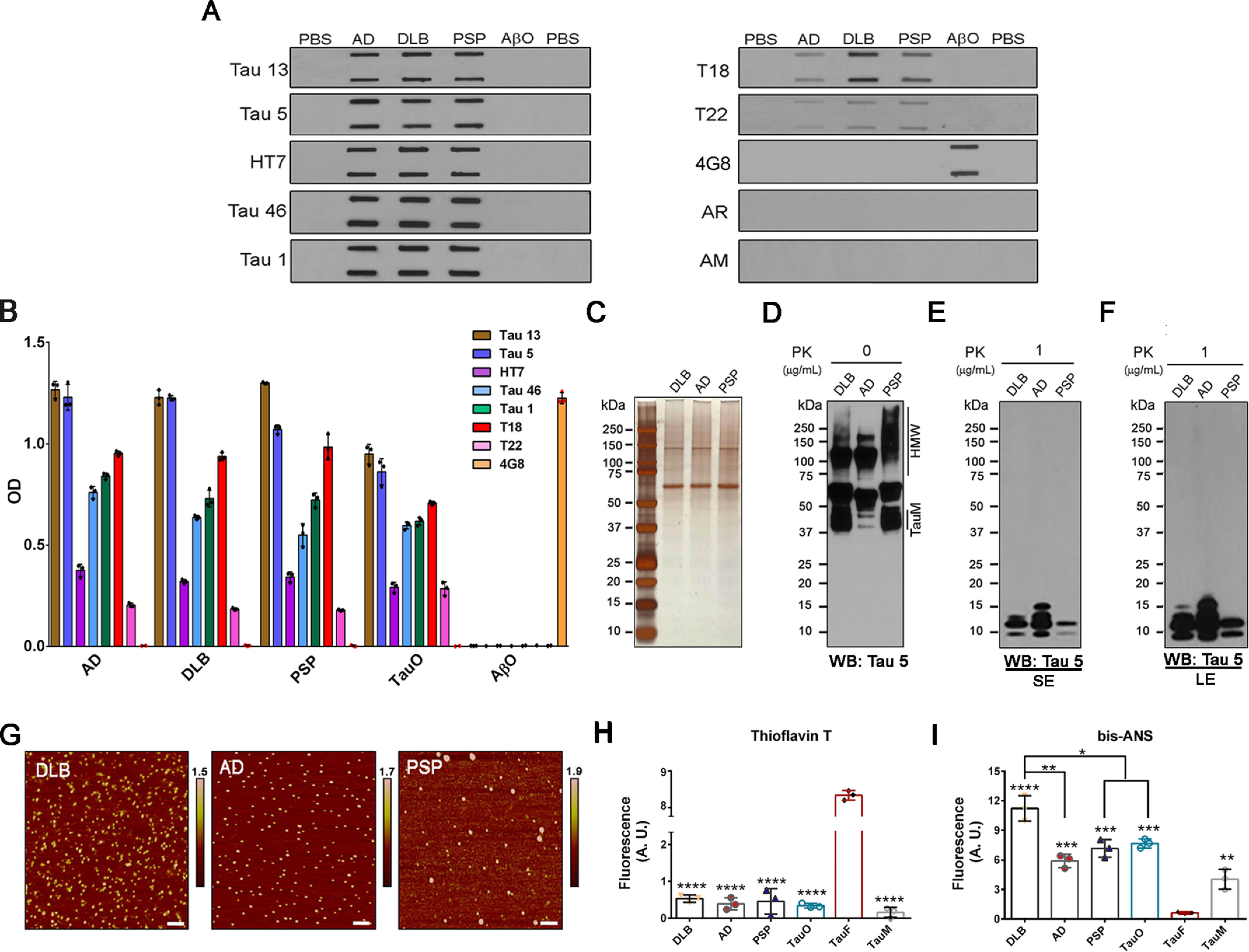
**Characterization of disease-relevant BDTOs.** *A*, filter trap assay analyses of BDTOs. PBS (buffer used for the BDTOs) and recombinant AβO were used as negative controls. Equal amounts of samples were loaded and probed with generic total tau antibodies, Tau 13, Tau 5, HT7, Tau 46, and Tau 1; T18, which recognizes misfolded tau; the oligomeric-specific tau antibody, T22; and the control anti-Aβ antibody, 4G8. Anti-mouse (*AM*) and anti-rabbit (*AR*) antibodies were used to exclude nonspecific probe binding to the filter membrane. *B*, ELISAs of oligomeric tau probed with generic total tau antibodies, Tau 13, Tau 5, HT7, Tau 46, and Tau 1; T18, which recognizes misfolded tau; the oligomeric-specific tau antibody, T22; and the anti-Aβ antibody, 4G8. Recombinant TauO and AβO were used as positive and negative controls, respectively. *C*, representative SDS-PAGE of BDTOs visualized by silver staining. *D–F*, representative WB images of BDTOs, without (*D*) or with 1 μg/ml PK enzyme (*E* and *F*), probed with the sequence-specific tau antibody, Tau 5. Short exposure (*SE*) and long exposure (*LE*) of immunoblots are presented in *E* and *F*, respectively, showing that the differential patterns of fragmentation are maintained with increasing time of exposure. *G*, representative AFM images of BDTOs isolated from different tauopathies. *Scale bars*, 100 nm. *H*, fluorescence intensity measurement of ThT to BDTOs and controls, including TauO, TauF, and TauM. *I*, fluorescence intensity measurement of bis-ANS to BDTOs and controls (TauO, TauF, and TauM). ThT + PBS and bis-ANS + PBS were used for background correction in *H* and *I*, respectively. Data in *H* and *I* were compared by one-way ANOVA followed by Dunnett's multiple-comparison test: *, *p* < 0.05; **, *p* < 0.01; ***, *p* < 0.001; ****, *p* < 0.0001. *Bars* and *error bars*, mean ± S.D.

First, BDTOs and appropriate control samples were biochemically evaluated by filter trap assay (FTA) and ELISA (Fig. 1, *A* and *B*) using a panel of widely used commercially available sequence-specific anti-tau antibodies (Tau 13, Tau 1, HT7, Tau 5, and Tau 46), which recognize N-terminal, mid-domain, and C-terminal regions of tau protein, along with in-house tau antibodies, T18 and T22, that recognize misfolded tau aggregates and tau oligomers, respectively (49, 52). 4G8 antibody was used for the detection of β-amyloid oligomers (AβO), which we used as negative control, and anti-mouse and anti-rabbit antibodies were used to exclude the nonspecific binding to the membrane. The FTA results showed differences in BDTO immunoreactivity with T18 and T22 but not the generic tau antibodies (Fig. 1*A*). AD BDTOs showed reduced immunoreactivity to T18 and T22 compared with DLB and PSP BDTOs, and PSP BDTOs showed decreased T18 immunoreactivity compared with DLB BDTOs (Fig. 1*A*). In both FTA and ELISA assays, 4G8 showed strong immunoreactivity with AβO but not with BDTOs, which suggests the absence of Aβ in the isolated tau oligomers.

Next, BDTOs were resolved by SDS-PAGE and visualized by silver staining (Fig. 1*C*). One of the most common determinants of prion strain differences is the stability of the protein core following exposure to proteinase K (PK) enzyme (53, 54). Recent studies demonstrated that prion-like tau aggregates also exhibit differential protease stability (19, 55, 56). To evaluate conformational differences within brain-derived tau oligomers, samples were exposed to 0 and 1 μg/ml PK and evaluated by Western blotting (WB) using the sequence-specific anti-tau antibody Tau 5 (Fig. 1, *D–F*). WB analysis of undigested (0 μg/ml PK) and digested samples (1 μg/ml PK) showed that BDTOs were sensitive to PK enzyme, revealing a different pattern of fragmentation of each BDTO following proteolytic digestion with PK (Fig. 1, *D–F*). Undigested samples exhibited a different profile of Tau 5 immunoreactivity with different high-molecular weight (HMW; 75–250 kDa) tau oligomers in AD, DLB, and PSP (Fig. 1*D*). Following PK digestion, DLB oligomeric tau showed three bands within molecular weights of 10–15 kDa, AD had four fragmentation bands, and PSP exhibited two bands (Fig. 1, *E* and *F*). The differential digestion profile remained unaltered with increasing time of exposure, as shown by short exposure (*SE*) (Fig. 1*E*) and long exposure (*LE*) (Fig. 1*F*). The different pattern of fragmentation is indicative of conformation differences that may account for different seeding potency and toxicity of disease-relevant tau oligomers.

In addition, the morphology of BDTOs was evaluated using atomic force microscopy (AFM). AFM images of oligomeric tau aggregates showed their classical homogeneous spherical morphology (Fig. 1*G*). Moreover, to gain more insight into the aggregation state and hydrophobicity of BDTOs, thioflavin T (ThT) and 4,4′-dianilino-1,1′-binaphthyl-5,5′-disulfonic acid, dipotassium salt (bis-ANS) fluorescence binding assays were carried out with BDTOs and control samples, including recombinant tau oligomers (TauO), tau fibrils (TauF), and TauM (Fig. 1, *H* and *I*). As expected, hydrophobic BDTOs and recombinant TauO and TauM were shown to bind weakly to ThT as compared with the TauF, used as the positive control (Fig. 1*H*). On the other hand, bis-ANS spectroscopic analyses showed that BDTOs and TauO have significantly higher binding affinity to bis-ANS as compared with TauF and TauM (Fig. 1*I*). Furthermore, brain-derived tau oligomers from DLB showed strong binding to bis-ANS as compared with AD and PSP BDTOs. Altogether, these results showed that disease-relevant tau oligomers isolated from AD, DLB, and PSP form structurally distinct conformers.

### Characterization of BDTOs in the presence of curcumin derivatives

We recently showed that synthetic curcumin derivatives interact with TauO, resulting in the formation of larger tau aggregates with decreased associated neurotoxicity (48). These novel curcumin derivatives are characterized by the removal of the β-diketo moiety that exhibits keto-enol tautomerism and is thought to be responsible for the low curcumin brain bioavailability (48).

**Figure 2.**
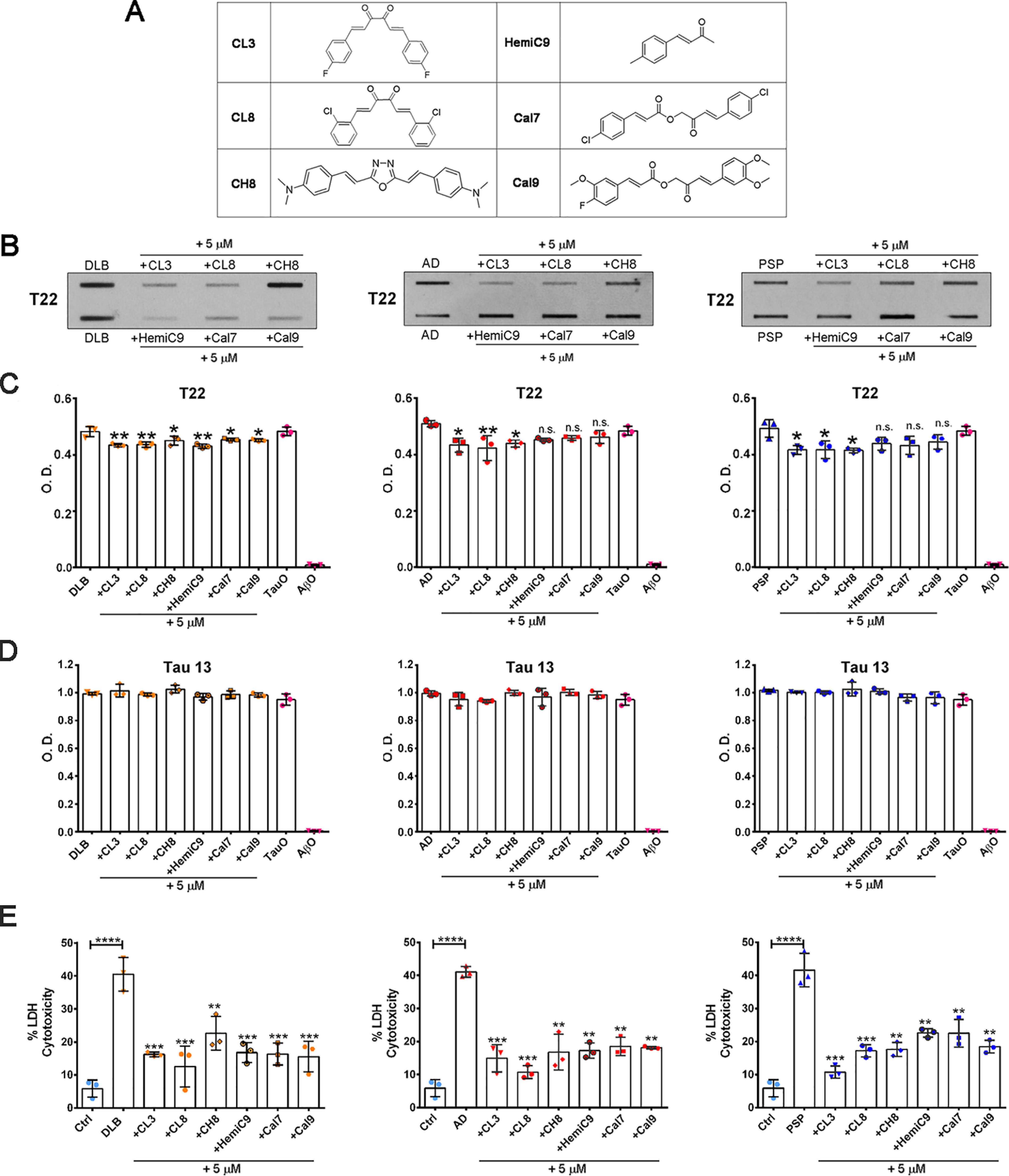
**Biochemical characterization and cytotoxicity profile of BDTOs with and without curcumin derivatives.** *A*, structure of newly synthesized curcumin derivatives. *B*, FTA analyses of BDTOs, alone and pretreated with curcumin derivatives (final concentration 5 μm). Equal amounts of samples were loaded and probed with the oligomeric-specific tau antibody, T22. *C*, indirect ELISA of BDTOs, alone and pretreated with curcumin derivatives, probed with anti-oligomeric tau antibody, T22. *D*, indirect ELISA of BDTOs, alone and pretreated with curcumin derivatives, probed with the sequence-specific tau antibody, Tau 13. TauO and AβO were used as positive and negative controls, respectively, in *C* and *D*. *E*, cytotoxic effect of 0.5 μm DLB, AD, and PSP BDTOs, alone or in the presence of curcumin derivatives (CL3, CL8, CH8, HemiC9, Cal7, and Cal9; final concentration 5 μm), on primary neurons for 24 h as measured by LDH release. Each experiment was performed in triplicate (*n* = 3). Data in *C–E* were compared by one-way ANOVA followed by Dunnett's multiple-comparison test: *, *p* < 0.05; **, *p* < 0.01; ***, *p* < 0.001; ****, *p* < 0.0001. *Bars* and *error bars*, mean ± S.D.

In this study, the six selected promising compounds (Fig. 2*A*) were screened against disease-relevant BDTOs. Therefore, BDTOs were incubated with and without curcumin derivatives (final concentration 5 μm) for 16 h on an orbital shaker at room temperature, under oligomerization conditions, and evaluated biochemically using the anti-oligomeric tau antibody T22 as well as a generic tau antibody, Tau 13. FTA analyses revealed that some compounds interact with BDTOs, resulting in decreased oligomer levels as shown by the reduced T22 immunoreactivity (Fig. 2*B*). In the FTA, larger aggregates are filtered through the membrane that traps and retains large protein aggregates, whereas small species, including protein monomers, can pass through (Fig. 2*B*). Moreover, indirect ELISA confirmed that the interactions of the six curcumin derivatives with DLB BDTOs reduced oligomeric levels, as seen by the decreased T22 immunoreactivity, whereas not all the compounds decreased the T22 signal in AD and PSP BDTOs (Fig. 2*C*). As expected, no changes were observed in total tau levels detected by Tau 13 immunoreactivity in ELISA analysis (Fig. 2*D*).

Furthermore, the toxicity of BDTOs, alone or pretreated with curcumin derivatives, was evaluated by a lactate dehydrogenase (LDH)-based assay (Fig. 2*E*). We have previously shown that the selected curcumin analogs have no toxic effects at 5 μm concentration in primary cortical neurons as assessed by LDH and MTS assay over the course of 24 h, at three time points: 2, 12, and 24 h (48). Therefore, primary cortical neurons, isolated from embryos of C57BL/6, were exposed to 0.5 μm BDTOs alone or BDTOs pretreated with 5 μm curcumin derivatives for 24 h. LDH analyses showed cytotoxicity of BDTOs, as the amount of LDH released in the cell culture media from damaged primary neurons is indicative of cellular cytotoxicity and cytolysis (Fig. 2*E*). Interestingly, primary neurons exposed to BDTOs pretreated with the curcumin derivatives showed a significantly lower LDH release as compared with those exposed to untreated BDTOs (Fig. 2*E*). Altogether, these results suggest that the selected curcumin derivatives modulate the aggregation pathways of BDTOs and rescue neurons from BDTO-induced cytotoxicity.

**Figure 3.**
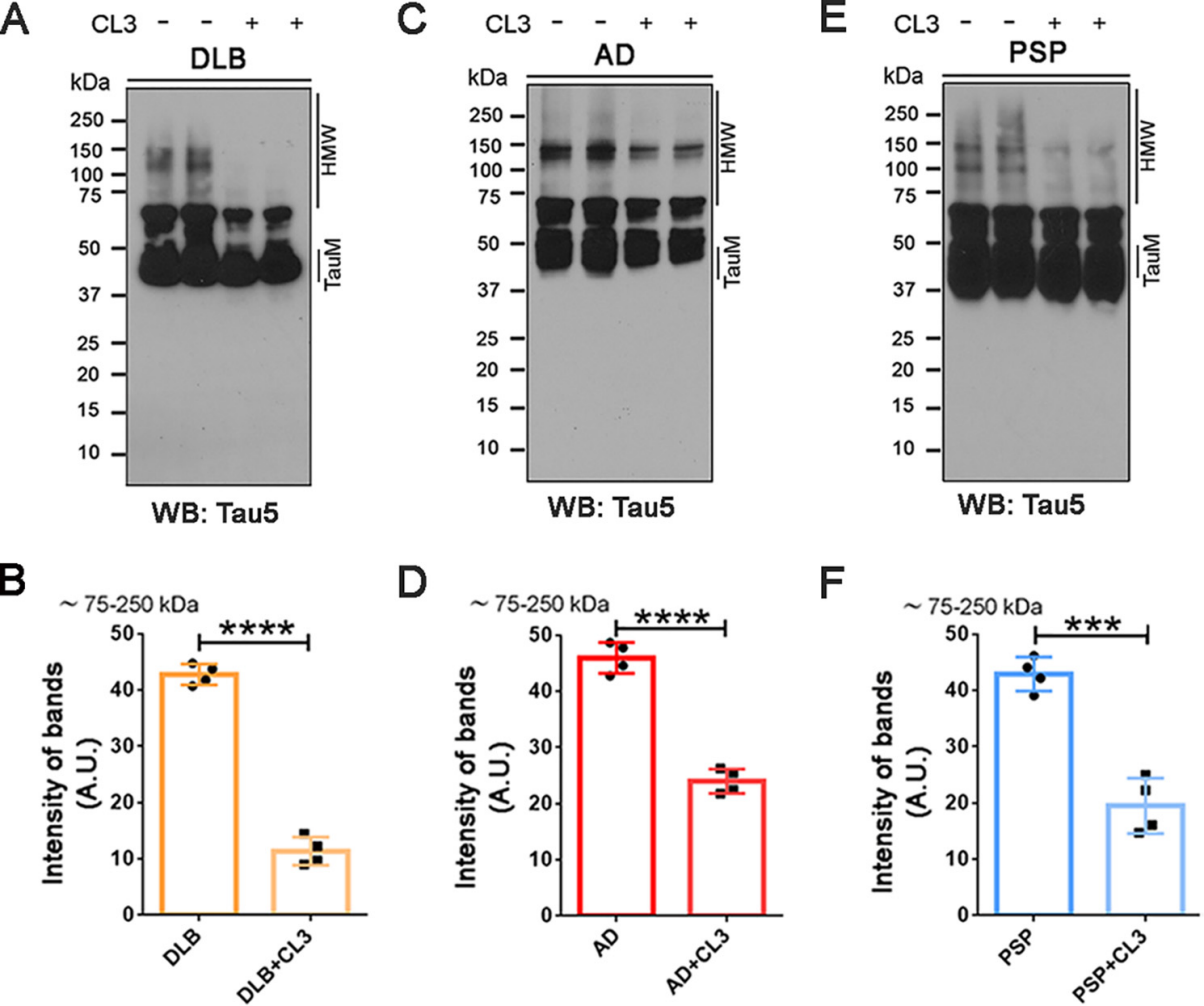
**Immunological detection of CL3-induced brain-derived tau aggregates.** *A*, representative WB images of DLB BDTOs alone (−) and pretreated (+) with CL3 and probed with the generic tau antibody, Tau 5. *B*, Tau 5 intensity of the 75 kDa and above bands (75–250 kDa) of DLB BDTOs untreated and pretreated with CL3. *C*, representative WB images of AD BDTOs alone (−) and pretreated (+) with CL3 and probed with the generic tau antibody, Tau 5. *D*, Tau 5 intensity of the 75 kDa and above bands (75–250 kDa) of AD BDTOs untreated and pretreated with CL3. *E*, representative WB images of PSP BDTOs alone (−) and pretreated (+) with CL3 and probed with the generic tau antibody, Tau 5. *F*, Tau 5 intensity of the 75 kDa and above bands (75–250 kDa) of PSP BDTOs untreated and pretreated with CL3. Samples are presented in duplicate in *A*, *C*, and *E*. Data in *B*, *D*, and *F* were analyzed by Student's *t* test: ***, *p* < 0.001; ****, *p* < 0.0001. *Bars* and *error bars*, mean ± S.D.

Among the six derivatives, CL3 and CL8 were found to modulate AD, DLB, and PSP oligomeric tau more efficiently than the others. For our successive analyses of the BDTOs modulated by curcumin derivatives, we chose CL3, which consistently showed high activity throughout the *in vitro* and cell-based assays. Therefore, the end products of the reaction of AD, DLB, and PSP BDTOs preincubated with 5 μm CL3 were resolved in a SDS-PAGE, followed by WB analysis using Tau 5 antibody, which showed a different pattern of immunoreactivity for each type of BDTO (Fig. 3, *A*, *C*, and *E*). Indeed, DLB BDTOs showed a distinctive band at 100 kDa (Fig. 3*A*), whereas AD BDTOs displayed two bands at ∼120–150 kDa (Fig. 3*C*), and PSP BDTOs showed two characteristic bands at ∼100 and ∼150 kDa (Fig. 3*E*). Interestingly, following incubation with CL3, we observed a significant decrease of these HMW tau aggregates as shown in the Tau 5 quantification for DLB (Fig. 3*B*), AD (Fig. 3*D*), and PSP brain–derived tau oligomers (Fig. 3*F*).

### Morphological characterization of CL3-induced brain-derived tau aggregates

**Figure 4.**
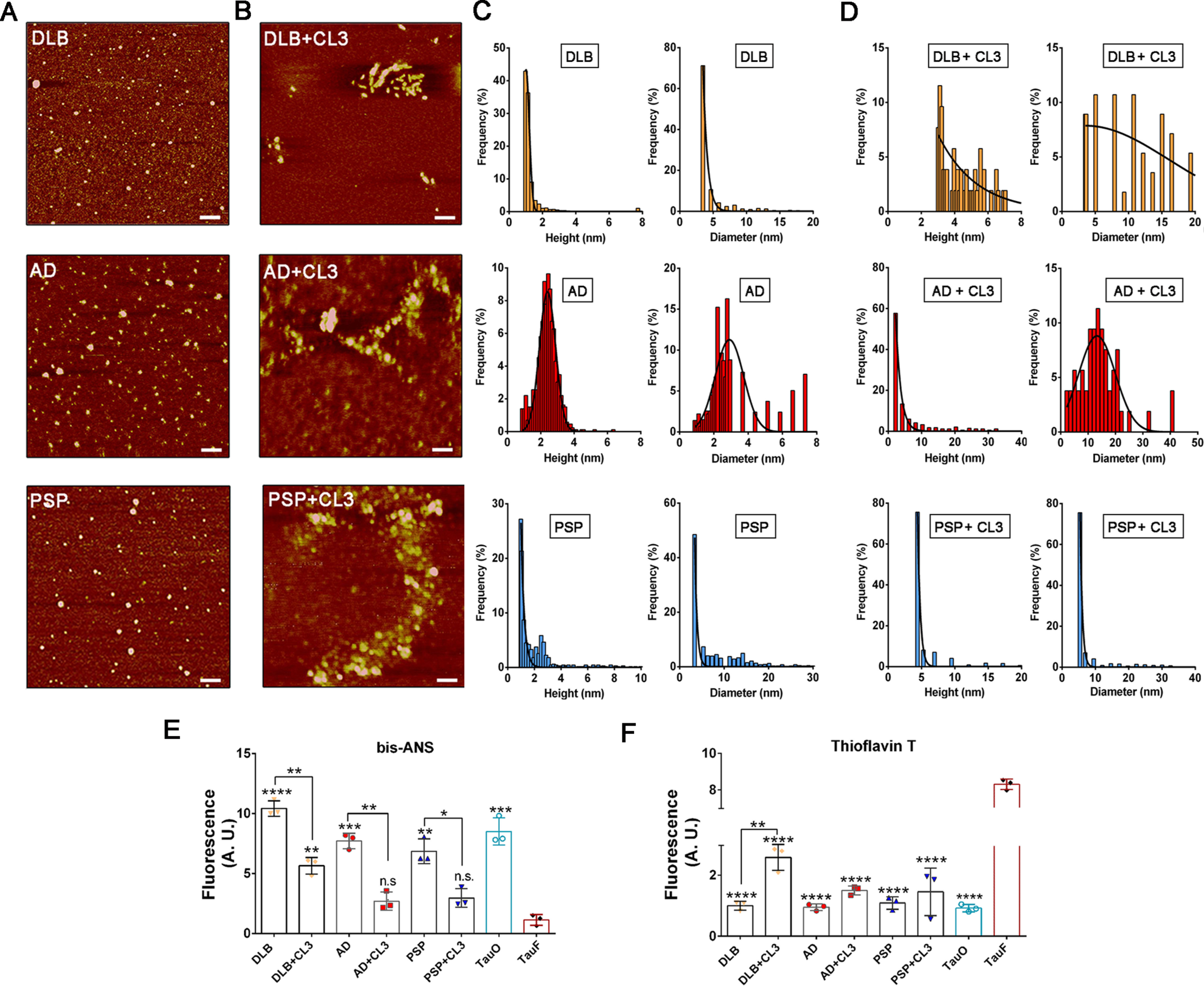
**Morphological characterization of BDTOs with and without CL3.** *A* and *B*, representative AFM images of BDTOs without (*A*) or with preincubation with 5 μm of CL3 (*B*); *scale bars*, 100 nm. *C*, height and diameter distribution histograms of DLB, AD, and PSP BDTOs. The height mean ± S.D. for each sample were as follows: DLB, 1.199 ± 0.5628; AD, 2.418 ± 0.6024; PSP, 1.755 ± 1.429. Diameter mean ± S.D. for each sample were as follows: DLB, 4.442 ± 2.247; AD, 2.908 ± 0.8286; PSP, 7.499 ± 5.636. *D*, height and diameter distribution histograms of DLB, AD, and PSP BDTOs pretreated with CL3. Height mean ± S.D. for each sample were as follows: DLB + CL3, 4.421 ± 1.324; AD + CL3, 5.966 ± 7.145; PSP + CL3, 5.403 ± 2.750. Diameter mean ± S.D. for each sample were as follows: DLB + CL3, 9.682 ± 5.066; AD + CL3, 13.27 ± 6.711; PSP + CL3, 7.458 ± 5.593. *E*, fluorescence intensity measurement of bis-ANS to BDTOs in the presence and absence of CL3. TauO and TauF were used as positive and negative controls, respectively. *F*, fluorescence intensity measurement of ThT to BDTOs alone and in the presence of CL3. TauO and TauF were used as negative and positive controls, respectively. Data in *E* and *F* were compared by one-way ANOVA followed by Dunnett's multiple-comparison test: *, *p* < 0.05; **, *p* < 0.01; ***, *p* < 0.001; ****, *p* < 0.0001. *Bars* and *error bars*, mean ± S.D.

To gain more insight into the structure and distribution of tau aggregates upon treatment with CL3, BDTOs with or without CL3 were morphologically defined by AFM (Fig. 4, *A* and *B*). Examination of untreated BDTOs by AFM revealed the classical spherical morphology of tau oligomers as shown by their respective height and diameter distribution histograms (Fig. 4*C*). AFM analyses of all the BDTOs preincubated with CL3 mostly showed clusters and/or larger tau aggregates, which was confirmed by their height and diameter distribution (Fig. 4, *B–D*). Notably, following incubation with CL3, we observed an increased height of DLB, AD, and PSP tau aggregates (Fig. 4*D*). In addition, the diameter of DLB and AD tau oligomers also increased after treatment with CL3, whereas no such changes were observed in PSP tau aggregates following incubation with CL3 (Fig. 4*D*).

Next, we performed the bis-ANS and ThT fluorescence binding assays with BDTOs and BDTOs preincubated with CL3 (Fig. 4, *E* and *F*). We observed decreased bis-ANS binding intensity for all of the three BDTOs incubated with CL3 as compared with the BDTOs alone (Fig. 4*E*). ThT did not show any significant fluorescence binding intensity to either the untreated BDTOs or BDTOs + CL3, except for BDTOs from DLB treated with CL3, which showed a higher ThT-binding affinity as compared with the untreated DLB (Fig. 4*F*). Recombinant TauO and TauF were used as controls, and all the readings were corrected for the background fluorescence to exclude any intrinsic fluorescence generated by the compound with either bis-ANS or ThT.

It has been shown that larger amyloid assemblies tend to have a lower ratio of surface to volume associated with a lower toxic effect compared with the smaller aggregates (57–59). In our study, we observed that the BDTOs exhibited an increase in size and morphology and a decrease in bis-ANS–binding intensity following incubation with CL3. We speculate that the CL3 has modulated the aggregation state of the BDTOs, which may display a lower surface to volume ratio, resulting in lower bis-ANS binding.

### CL3 rescues primary neurons from BDTO-associated neurotoxicity

**Figure 5.**
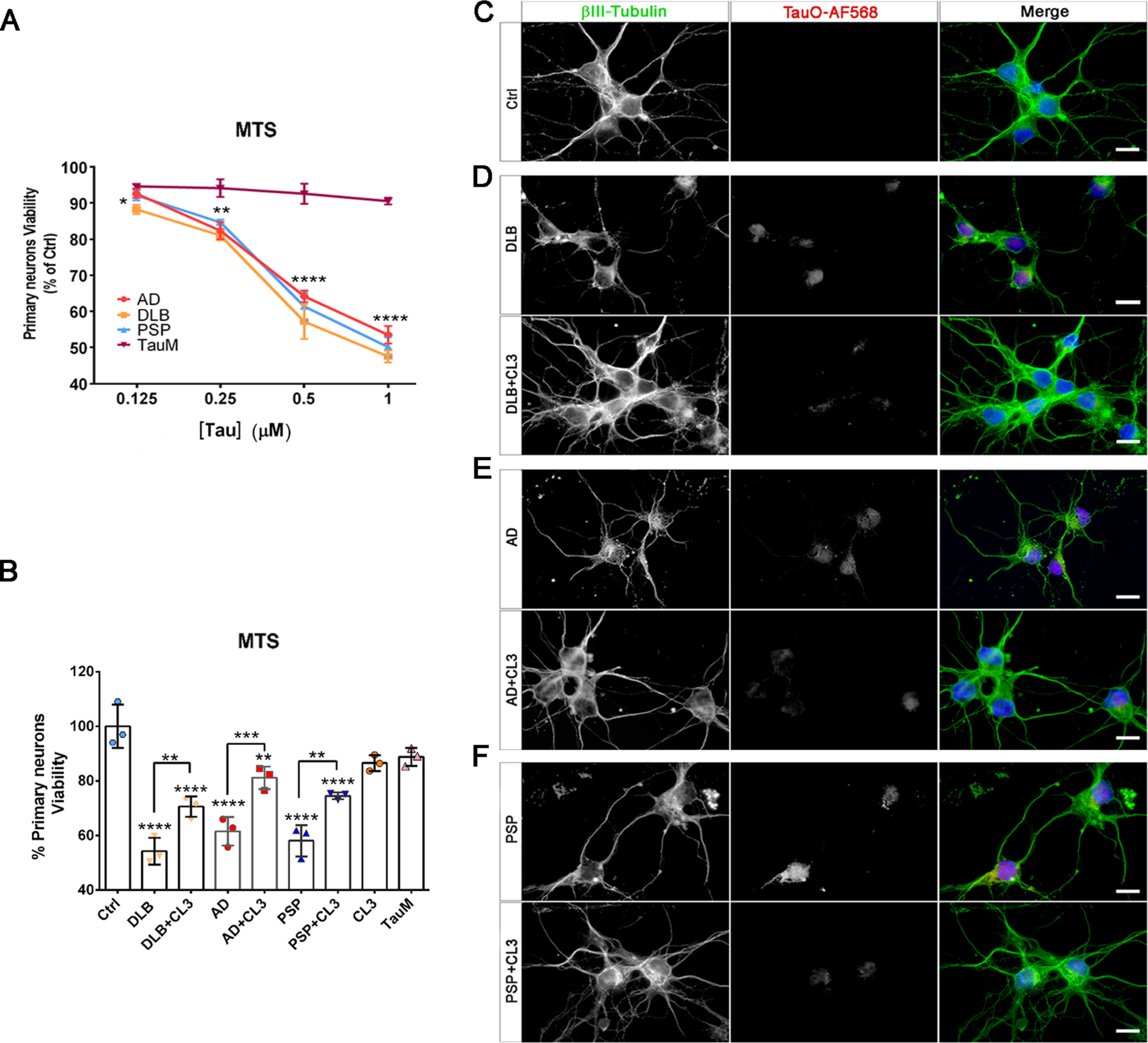
**Protective effect of CL3 on BDTOs-induced neurotoxicity in primary cortical neurons.** *A*, dose-dependent cell viability of primary neuronal culture exposed to BDTOs for 24 h. *B*, viability percentage of neuronal culture exposed to 0.5 μm BDTOs, 0.5 μm BDTOs preincubated with 5 μm CL3, CL3 alone, or TauM as a negative control for 24 h assessed by MTS assay. Each experiment was performed in triplicate (*n* = 3). *C–F*, representative epifluorescence images of untreated cortical neurons (*C*) and cortical neurons exposed to BDTOs labeled with AF568 or BDTOs labeled with AF568 in the presence of CL3 (*D–F*). Neurons were immunostained with βIII–Tubulin (*green* in *merge*), TauO (*red* in *merge*), and DAPI (*blue* in *merge*). *Scale bar*, 10 μm. Data in *A* and *B* were compared by one-way ANOVA followed by Dunnett's multiple-comparison test: *, *p* < 0.05; **, *p* < 0.01; ***, *p* < 0.001; ****, *p* < 0.0001. *Bars* and *error bars*, mean ± S.D.

An inverse correlation between the toxic effects of amyloid aggregates and their size as well as their surface hydrophobicity has been documented previously (57–59). Therefore, we investigated whether the CL3-induced tau aggregates have less toxic properties compared with the untreated BDTOs. The BDTO-associated toxicity was assessed using primary cortical neurons. Primary neurons were exposed to increasing concentrations of AD, DLB, and PSP tau oligomers (0.125–1 μm) for 24 h. The toxicity of BDTOs was evaluated using MTS assay, showing a dose-dependent toxicity of BDTOs. Indeed, cell viability significantly decreased at 0.25 μm BDTOs, reaching 60% at 0.5 μm (Fig. 5*A*).

Next, the toxic profiles of the CL3-induced tau aggregates were assessed by MTS assay in primary neurons using 0.5 μm BDTOs or BDTOs pretreated with 5 μm CL3 for 24 h. The cell viability significantly decreased after treatment with BDTOs, whereas pretreatment with CL3 significantly increased cell viability (Fig. 5*B*). We previously showed that 5 μm CL3 was able to recover primary cortical neurons from toxic recombinant tau oligomers (48). Nevertheless, in the current study, 5 μm CL3 showed a partial recovery of cell viability from toxic BDTOs, indicating that an increased concentration of CL3 and/or incubation time with the BDTOs might be needed to obtain a complete recovery from cytotoxicity. TauM and CL3 alone were used as negative controls showing a low toxicity profile as compared with the toxic BDTOs alone (Fig. 5*B*).

In addition, to further confirm our findings and gain a better understanding of the protective role of CL3, an internalization screening was carried out in primary neurons. Cortical neurons were exposed to 0.5 μm BDTOs labeled with Alexa Fluor™ (AF)568, or BDTOs labeled with AF568 pretreated with CL3, for 1 h (Fig. 5, *C–F*). Neurons were immunostained with βIII-Tubulin (a marker for mature neurons) and DAPI (for nuclei) and imaged by epifluorescence microscopy. Interestingly, BDTOs were observed in the cell bodies and projections of neurons, indicating that disease-relevant tau oligomers were extensively internalized by the neurons. Furthermore, βIII-Tubulin disruption was observed at the concentration used for the treatment with BDTOs, indicative of their toxic effects. On the other hand, immunofluorescence images of neurons treated with BDTOs pretreated with CL3 showed a decreased area positive to tau oligomer staining and recovery of neuronal cultures as seen by the βIII-Tubulin signal, comparable with that of the untreated control (Fig. 5, *C–F*).

**Figure 6.**
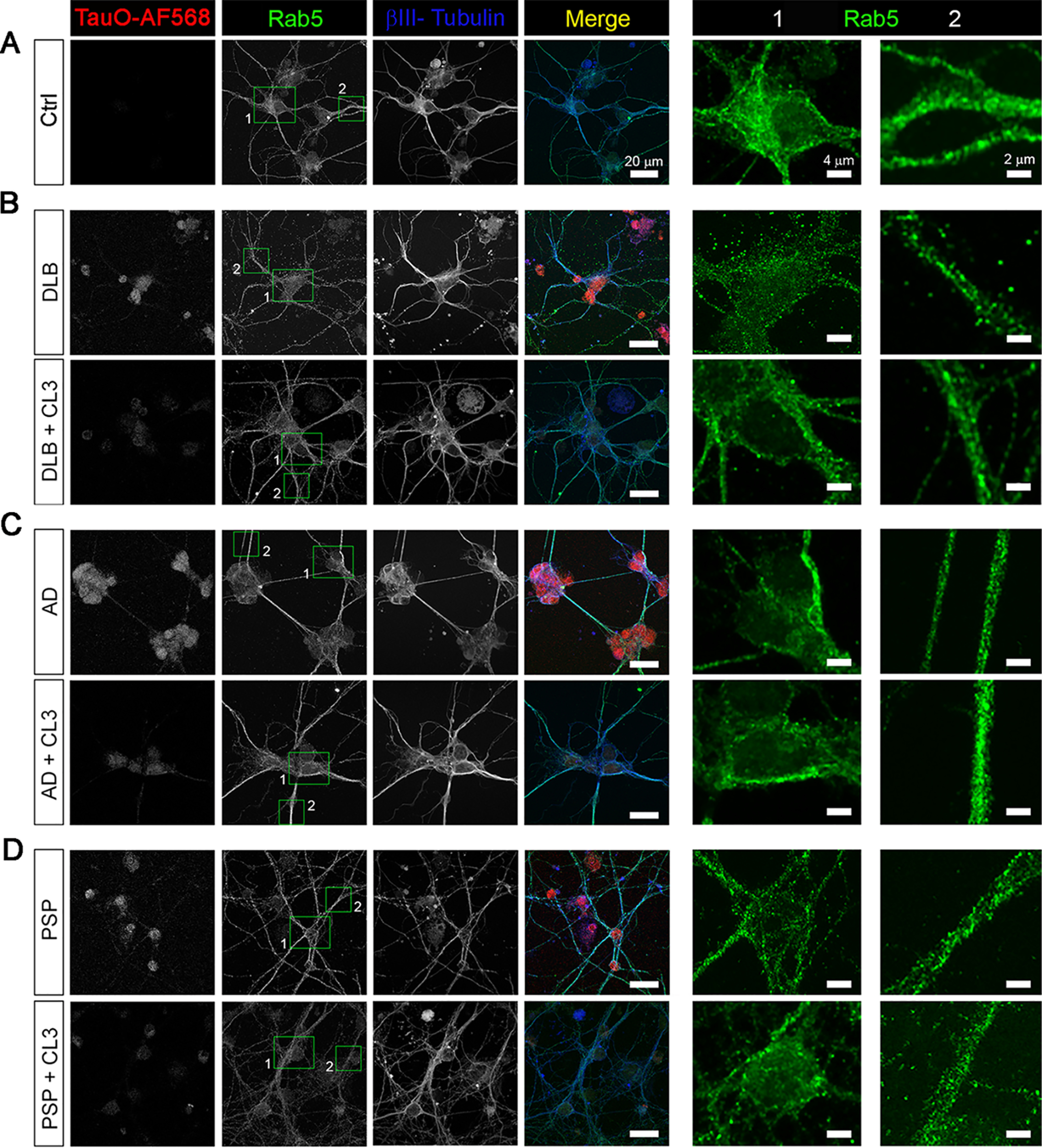
**Internalization of BDTOs in the absence and presence of CL3.** *A–D*, representative confocal images of untreated primary cortical neurons (*A*) or primary neurons exposed to 0.5 μm BDTOs labeled with AF568 or 0.5 μm BDTOs labeled with AF568, preincubated with 5 μm CL3. *B–D*, neurons were immunostained with βIII–Tubulin (*blue* in *merge*), TauO (*red* in *merge*), and Rab5 (*green* in *merge* and *insets*). *Scale bar*, 20 μm. *Zoomed images* of Rab5 (*green*) for cell body and neuronal projections are presented in *insets 1* and *2*, respectively. *Scale bar*, 4 μm (*inset 1*) and 2 μm (*inset 2*).

Furthermore, to gain more insight into the internalization process, we immunostained primary neurons with anti-Rab5 antibody, which is an early endosomal marker protein (Fig. 6). Indeed, Rab proteins are involved in the regulation of intracellular transport and the progression of the cargo through the endosomal system (60). Therefore, AF568-labeled BDTOs, or AF568-labeled BDTOs pretreated with CL3, were exogenously added to the neuronal culture and immunostained with anti-Rab5 and βIII-Tubulin antibodies. Confocal images showed different patterns of distribution of Rab5 signal in neurons exposed to DLB and PSP oligomeric tau (Fig. 6, *B* and *D*). Rab5-positive vesicles were distributed in a punctate pattern in the cell body as well as in the neuronal projections of DLB or PSP tau oligomer–treated neurons as shown in *zoomed insets 1* and *2*, respectively (Fig. 6, *B* and *D*). Neurons treated with AD oligomeric tau showed a linear distribution of Rab5 signal in the cell body (*inset 1*) with bright puncta observed in the neuronal projections (*inset 2*) (Fig. 6*C*). On the other hand, neurons exposed to BDTOs, pretreated with CL3, exhibited a diffused distribution comparable with that of the untreated control (Fig. 6*A*). These results suggest that CL3-induced tau aggregates are less prone to be internalized by the neurons, thus confirming their reduced associated neurotoxicity observed by MTS assay and immunofluorescence analyses.

### BDTO strains exhibit distinct seeding activities

**Figure 7.**
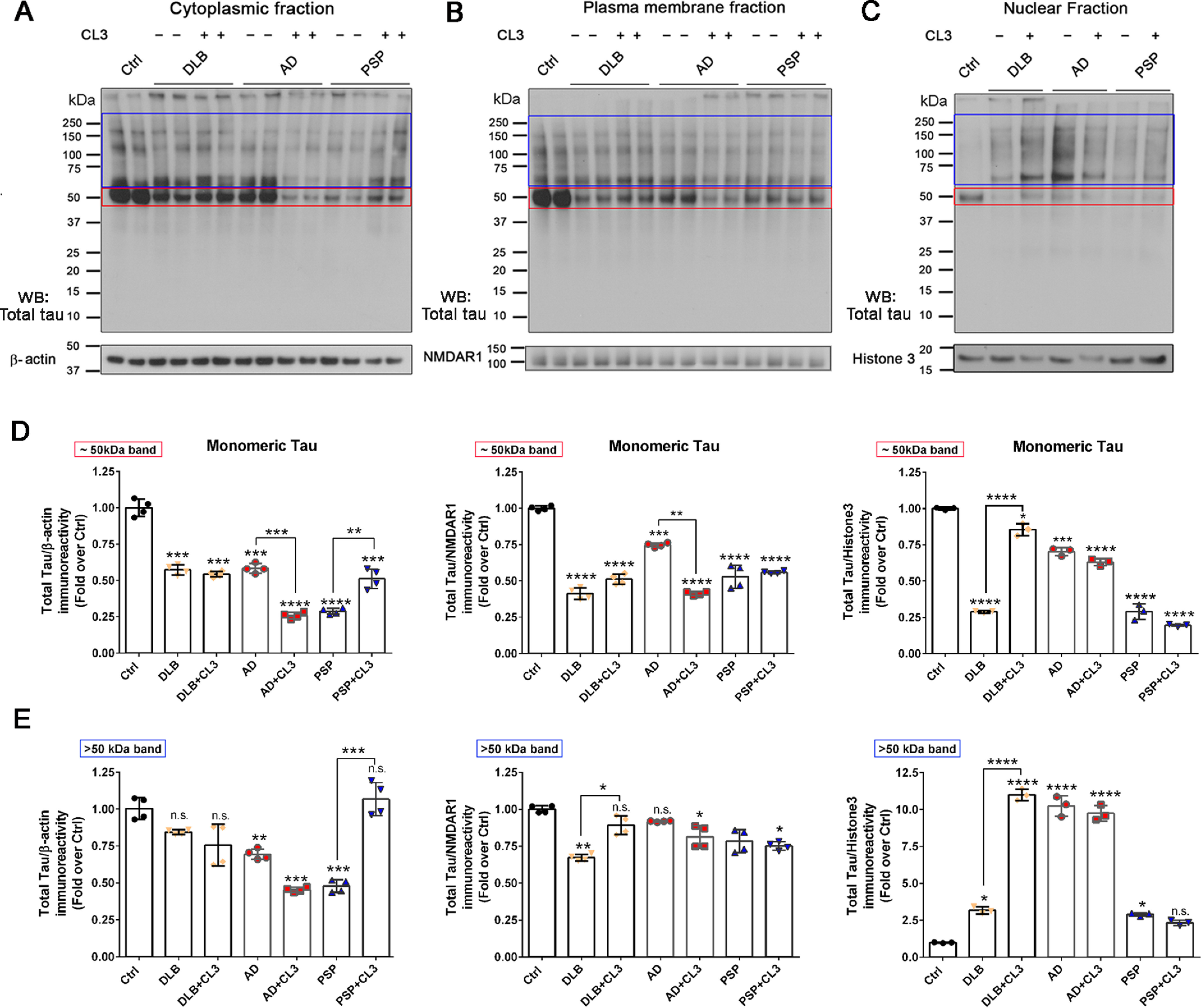
**Disease-relevant brain-derived tau oligomers with and without CL3 taken up by primary cortical neurons.** *A–C*, primary cortical neurons were exposed to 0.25 μm DLB, AD, and PSP BDTOs in the presence (+) and absence (−) of 5 μm CL3 for 24 h. Tau levels were evaluated in the cytoplasmic (*A*), plasma membrane (*B*), and nuclear (*C*) fractions using the generic tau antibody (*Total tau*). β-Actin, NMDAR1, and histone 3 were used as loading and subcellular fractionation purity control for cytoplasmic, plasma membrane, and nuclear fractions, respectively. Samples in cytoplasmic and plasma membrane fractions were loaded in duplicate. *D*, densitometric quantification of total tau immunoreactivity of 50 kDa bands for cytoplasmic, plasma membrane, and nuclear fractions. *E*, densitometric quantification of total tau immunoreactivity of bands above 50 kDa for cytoplasmic, plasma membrane, and nuclear fractions. Data in *D* and *E* were compared by one-way ANOVA followed by Dunnett's multiple-comparison test: *, *p* < 0.05; **, *p* < 0.01; ***, *p* < 0.001; ****, *p* < 0.0001; *n.s.*, not significant. *Bars* and *error bars*, mean ± S.D.

Next, to acquire insight into the internalization and seeding activities and to investigate the effects of BDTOs on primary neurons, we performed subcellular fractionation of neuronal culture treated with 0.25 μm BDTOs, or BDTOs pretreated with CL3, for 24 h (Fig. 7). Tau levels in the cytoplasmic, plasma membrane, and nuclear fractions were evaluated by WB using a generic tau antibody that recognizes total tau (Fig. 7, *A–C*). β-Actin, NMDAR1, and histone 3 antibodies were used as controls for loading and purity of subcellular fractionations.

WB analysis showed that exogenous BDTOs seed for the tau aggregation in primary neurons as shown by the significantly decreased level of tau monomers in the cytoplasmic, plasma membrane, and nuclear fractions upon treatment with BDTOs (Fig. 7, *A–C*). Neurons treated with DLB BDTOs showed a decrease of endogenous tau monomers in the cytoplasmic and plasma membrane fractions, and a more significant reduction in the nuclear fraction was observed as compared with the untreated neurons (*Ctrl*) (Fig. 7*D*). AD BDTO-treated neurons showed reduced monomeric tau in the plasma membrane and nuclear fractions with a more pronounced decrease in the cytoplasmic compartment (Fig. 7*D*). On the other hand, PSP tau oligomers showed a significant decrease in monomeric tau in the plasma membrane fraction and even more reduced tau monomers in the cytoplasmic and nuclear fractions (Fig. 7*D*).

The pretreatment of BDTOs with CL3 modulated the aggregation state of tau oligomers from AD, DLB, and PSP and affected their seeding activity in primary neurons. Noticeably, neurons exposed to AD BDTOs pretreated with CL3 showed decreased monomeric tau in the cytoplasmic and plasma membrane fractions as compared with untreated AD BDTOs, and no changes were observed in tau levels in the nuclear fraction (Fig. 7*D*). On the other hand, neurons treated with DLB BDTOs, pretreated with CL3, displayed increased monomeric tau in the nuclear fraction as compared with those neurons treated with untreated DLB BDTOs and comparable with the untreated neurons. Furthermore, neurons exposed to DLB BDTOs, pretreated with CL3, showed the formation of larger tau aggregates as shown by quantifying the total tau signal above the 50 kDa band (Fig. 7*E*). Neurons exposed to PSP BDTOs pretreated with CL3 showed increased monomeric tau as well as HMW aggregates in the cytoplasmic fraction as compared with PSP BDTO-treated neurons (Fig. 7, *D* and *E*). Notably, CL3-induced PSP tau aggregates restored the pattern of immunoreactivity of endogenous tau in the cytoplasmic fraction, as shown by the quantification of total tau intensity above the 50 kDa band (Fig. 7*E*). No significant changes were observed between neurons treated with PSP BDTOs and CL3-induced PSP tau aggregates in the plasma membrane and nuclear fractions (Fig. 7, *D* and *E*).

Altogether, these results suggest that exogenous BDTOs from different diseases induce tau seeding in primary cortical neurons, leading to the formation of tau aggregates that are different in morphology and their cellular distributions. Significant differences are observed within BDTO strains and within cell compartments evaluated by total tau immunoreactivity, suggesting a strain-specific effect for each cell compartment. The seeding effects of the BDTOs were affected differently by pretreatment with CL3. These results confirm that CL3 modulates the aggregation state of BDTOs, promoting the formation of new tau species as seen by their effects on endogenous tau in primary neurons.

**Figure 8.**
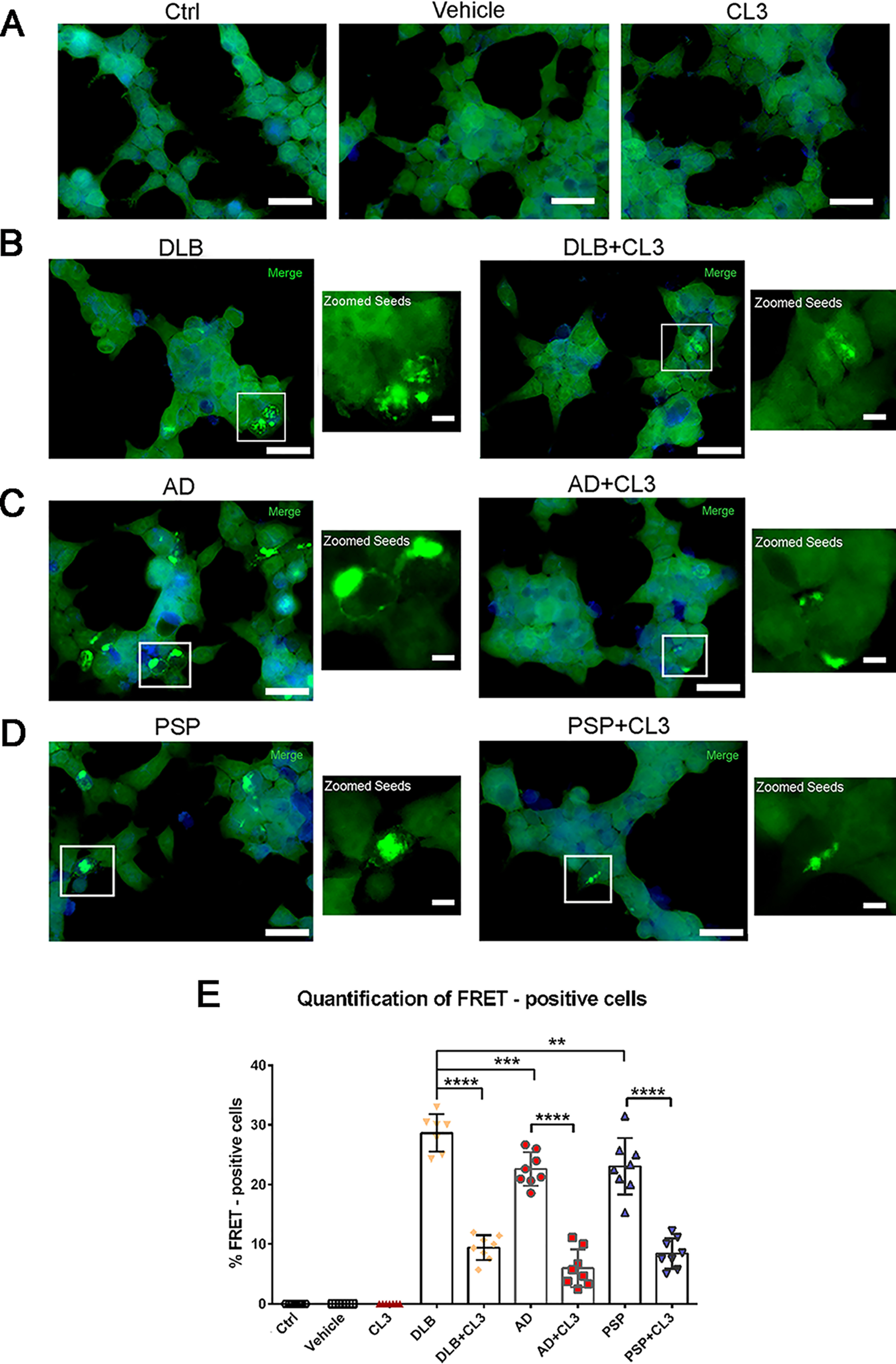
**Tau RD P301S biosensor cells treated with BDTOs in the presence or absence of CL3.** *A–D*, FRET-based HEK-Tau biosensor cells were used to determine the seeding activity of BDTOs isolated from human brain tissues. Cells were treated with Lipofectamine 2000 (vehicle) (*A*) or empirical concentration at 75 nm BDTO/liposome mixture in the presence and absence of 5 μm CL3 for 24 h (*B–D*). *Scale bar*, 20 μm. *Insets* are presented for biosensor cells exposed to BDTOs alone or in the presence of CL3. *Scale bar*, 20 μm. *E*, quantification of FRET-positive cells for each condition. Images were taken from ×40 magnification of three random fields in triplicate. The percentage of FRET-positive cells was calculated from total FRET-positive cells divided by total DAPI-positive cells. Data in *E* were compared by one-way ANOVA followed by Dunnett's multiple-comparison test. **, *p* < 0.01; ***, *p* < 0.001; ****, *p* < 0.0001. *Bars* and *error bars*, mean ± S.D.

In addition, we further investigated the seeding activity of AD, DLB, and PSP brain–derived oligomeric tau using the established FRET-based HEK-Tau biosensor cells. Tau aggregates from human and mouse brain lysates applied into Tau RD P301S biosensor cells were able to seed tau aggregation, forming intracellular tau inclusions observed by the appearance of FRET signal (19, 51, 61). We previously showed that BDTOs can seed for intracellular tau aggregation in the tau biosensor cells (51). Therefore, to examine whether CL3 has any effect on the seeding potencies of the BDTOs, we exogenously added BDTOs, or BDTOs pretreated with CL3, to the tau biosensor cells. The sublethal dose (75 nm) used for seeding the activity of the three distinct BDTO strains was empirically determined. Cells were exposed to exogenous tau seeds mixed with Lipofectamine 2000 to facilitate the transduction of the seeds into the cells for 24 h. The applied AD, DLB, and PSP oligomeric tau seeds, alone or in the presence of CL3, nucleated aggregation of tau as detected by immunofluorescence microscopy (Fig. 8). As expected, no FRET-positive cells were observed with liposome vehicle or liposome in the presence of CL3 or untreated control (Fig. 8*A*), whereas cells transduced with AD, DLB, and PSP oligomeric tau seeds exhibited a significant increase of FRET-positive cells as shown by the bright green signals (Fig. 8, *B–D*), indicating intercellular tau inclusion formation. DLB, AD, and PSP BDTOs showed prominent seeding activity and unique aggregation patterns as observed in the *zoomed images*. In addition, we observed that the pretreatment of BDTOs with CL3 affected the size and morphology of BDTOs tau aggregates and significantly decreased their seeding propensity (Fig. 8*E*).

Altogether, these results suggest that BDTOs, isolated from different tauopathies, possess different seeding potencies exhibiting different tau inclusion formation. In addition, our observations showed that CL3 affects the aggregation state of BDTOs, resulting in the formation of tau aggregates with a decreased seeding activity.

## Discussion

A growing body of evidence suggests that tau plays a causal role in mediating neurodegeneration and is strongly correlated with cognitive decline in AD and related diseases (62, 63). Tauopathies comprise a large group of age-related neurodegenerative diseases, characterized by predominant accumulation and deposition of pathological tau aggregates or in combination with other toxic amyloid aggregates (18, 23, 50, 64–67). Recent evidence has suggested that the accumulation of tau is mediated through the spreading of misfolded tau seeds from cell-to-cell and from initial brain regions throughout the brain in a trans-synaptic pattern as the disease progresses, thus proposing a prion-like mechanism for tau protein to propagate the disease (19, 68, 69). Furthermore, the association of diverse tau strains with different disorders suggests that they may be partly responsible for the diverse outcomes of tauopathies, explaining how the aggregation of the same protein can cause different diseases and diverse progression and phenotypes (61, 70–72).

To date, most of the tau strain studies have been performed using fibrillar tau (19, 20, 73); however, the mechanism by which the toxic tau oligomers induce seeding and propagation of tau pathology has not yet been specifically investigated. Indeed, the mechanism by which the toxic tau aggregates are propagated between cells is still unclear. Therefore, more investigations are needed to better understand how tau is released in the extracellular space to be then internalized into neighboring or anatomically connected cells and sequentially template for further aggregation within those cells (74, 75). It is well-known that pathogenic tau is taken up into the neuron (76, 77). Recent studies from our laboratory suggested that the heparan sulfate proteoglycan–mediated pathway regulates the internalization of BDTOs from AD and DLB, whereas BDTOs from PSP are taken up by heparan sulfate proteoglycan–mediated and other alternative pathways (51).

The conformational diversity of tau oligomers and the highly dynamic nature of these oligomeric strains, which can interconvert between many different assembly states or conformations, severely complicate efforts for developing therapeutic approaches for neurodegenerative diseases (16, 17). Treatment options should consider small molecules that are able to target, modulate, and stabilize toxic tau oligomeric strains in a nontoxic conformation or enhance the cellular protein quality control mechanisms, including autophagy and proteasomal degradation (8, 78–82). Modulating the conformations and depleting the disease-relevant structures using small molecules, including our novel curcumin analogs, could be an efficient therapeutic approach that targets their toxicity despite the many upstream factors that may be involved in the formation of tau oligomeric strains (48, 79, 80, 83, 84). Therefore, novel curcumin derivatives that have been previously shown to neutralize or modulate preformed toxic recombinant TauO (48) were screened and tested against well-characterized disease-relevant tau oligomeric strains isolated from AD, DLB, and PSP brain homogenates (23, 49–51, 64).

The characterization of disease-relevant tau strains is critical for the accurate study of tau pathology in disease as well as diagnostic and therapeutic applications (19, 70, 71, 85). Herein, we have thoroughly characterized disease-relevant tau oligomers and assessed their immunological, morphological, and biophysical characteristics. We have reported the existence of brain-derived tau oligomeric strains that can be differentiated by their sensitivity to PK enzyme digestion. Indeed, following exposure with PK, BDTOs exhibited differences in the protein core stability showing different cleavage patterns. These results suggest that conformational differences between BDTOs differentially affect the proteolytic digestion of tau aggregates.

Recent evidence has shown that both the size and the surface hydrophobicity of protein aggregates play a key role in their associated neurotoxicity (57–59). Indeed, there is an inverse correlation between the aggregates' toxicity and either their size or surface hydrophobicity (57–59). As the size of the amyloid aggregates increases, the ratio of their surface to volume decreases, and so do their toxic potencies (58, 59). Herein, we found that curcumin derivative CL3 interacts with disease-relevant tau oligomers, affecting their aggregation states by decreasing the oligomers' levels and promoting larger tau assembly formation. CL3-induced tau aggregates displayed a reduced binding to bis-ANS and increased size as revealed by AFM analyses. Therefore, in agreement with the previously mentioned studies and our previous studies using recombinant tau oligomers, our observations showed that the larger, less hydrophobic tau aggregates induced by CL3 are less prone to be internalized by the neurons and significantly less toxic. Finally, we showed that CL3-induced tau aggregates exhibit decreased seeding propensity in both primary cortical neurons and Tau RD P301S biosensor cells. Remarkably, the lowered internalization and seeding propensity of these larger and less hydrophobic tau aggregates support their reduced toxic effects.

Altogether, these results suggest that CL3 and other promising compounds could aid in the development of strain-specific novel therapeutic approaches for AD and related tauopathies. Additionally, CL3 is a fluorinated small molecule that can be easily synthesized using ^18^F for PET imaging. Therefore, CL3 could also serve as a molecular diagnostic probe for early intervention of the disease or identification of the dominant tau strains that may be more relevant for disease staging. Our findings lay the foundation for future studies to test the efficacy of CL3 *in vivo* using animal models of tauopathies and evaluate its potential for targeting tau oligomeric strains.

## Experimental procedures

### Chemistry

Compounds, synthesis, and characterization were reported previously (48). Compound chemical structure is provided in Fig. 2*A*.

### Biology

Animal handling and all the experimental procedures were performed in accordance with the Guide for the Care and Use of Laboratory Animals (National Institutes of Health) and according to the relevant guidelines and regulations approved by the Institutional Animal Care and Use Committee of the University of Texas Medical Branch (UTMB). Mice were housed at the UTMB animal care facility and maintained according to United States Department of Agriculture standards (12-h light/dark cycle with *ad libitum* access to water and food).

### Brain homogenate preparation

Post-mortem brain tissues from AD, DLB, and PSP were obtained from Oregon Health and Science University, the Institute for Brain Aging and Dementia (University of California, Irvine, CA, USA), and the Brain Resource Center at Johns Hopkins. Neuropathological assessment conformed to NIA/Reagan Institute, National Institutes of Health, consensus criteria. The following information was available for the cases used in this study: diagnosis, age at death, gender, post-mortem index, brain area, and Braak stage (Table 1). Each brain was homogenized in 1× PBS with a protease inhibitor mixture (Roche Applied Science, 11836145001), using a 1:3 dilution of brain/1× PBS (w/v). Samples were centrifuged at 10,000 rpm for 10 min at 4 °C. Supernatants were then aliquoted, snap-frozen, and stored at −80 °C until use.

**Table 1:** **List of human cases examined in this study**

Pathology/brain no.	Gender	Age (years)	Brain area	Post-mortem interval (h)	Braak stage
AD1	Male	77	Frontal cortex	4.5	VI
AD2	Female	82	Frontal cortex	3.1	VI
AD3	Male	83	Frontal cortex	3.5	VI
PSP1	Male	72	Frontal cortex	11	NA*^a^*
PSP2	Male	73	Frontal cortex	8.5	NA
PSP3	Male	79	Frontal cortex	12	NA
DLB1	Male	72	Frontal cortex	24	NA
DLB2	Female	67	Frontal cortex	12	NA
DLB3	Female	76	Frontal cortex	19	NA

*^a^* NA, not available.

### Immunoprecipitation of tau oligomers from human brain tissues

Immunoprecipitation experiments were performed as described previously (86). Briefly, tosyl-activated magnetic Dynabeads (Dynal Biotech, Lafayette Hill, PA, USA) were coated with 20 μg of T22 antibody (1.0 mg/ml) diluted in 0.1 m borate, pH 9.5, overnight at 37 °C. Beads were washed (0.2 m Tris, 0.1% BSA, pH 8.5) and then incubated with either AD, DLB, or PSP brain homogenate with rotation at room temperature for 1 h. Beads were then washed three times with PBS and eluted using 0.1 m glycine, pH 2.8. The pH of each eluted fraction was adjusted using 1 m Tris, pH 8.0. Fractions were then centrifuged in a micron centrifugal filter device with a molecular weight cut-off of 10 kDa (Millipore, 42415) at 14,000 × *g* for 25 min at 4 °C. Oligomers were then resuspended in sterile 1× PBS. Total protein concentration was determined using a Pierce^TM^ BCA protein assay kit (Thermo Scientific, 23225). The samples were again centrifuged in a micron centrifugal filter device with a cut-off of 10 kDa at 14,000 × *g* for 25 min at 4 °C. Oligomers were then resuspended in 1× PBS to obtain the desired concentration (0.1–0.5 mg/ml) and kept at −20 °C. No oligomers were found in the immunoprecipitate from control brains as reported previously by our group and others (22, 24).

### Preparation of recombinant tau species

Recombinant tau protein (Tau-441 (2N4R), 45.9 kDa) was expressed and purified as described (87, 88). The tau pellet was treated with 8 m urea followed by overnight dialysis against 1× PBS, pH 7.4. Tau concentration was measured using a Pierce^TM^ BCA protein assay kit (Thermo Scientific, 23225) and normalized to 1 mg/ml by adding 1× PBS. Aliquots of TauM in PBS were stored at −20 °C. Each 300 μl of tau stock (0.3 mg) was added to 700 μl of 1× PBS and incubated for 1 h on an orbital shaker at room temperature. After shaking, the resulting TauO were purified by FPLC (Superdex 200 Increase 10/300 column, Amersham Biosciences). Aliquots of TauM were incubated with heparin (15 kDa) (1:5 molar ratio) to prepare TauF at 37 °C on an orbital shaker at a speed of 30 rpm for 5 days as described previously (89).

### Fluorescence labeling of tau protein

BDTOs were labeled with AF568 NHS Ester (Invitrogen) according to the manufacturer's guidelines with minor modifications. Briefly, AF568 NHS Ester was dissolved in 100 mm sodium bicarbonate to make the final concentration 1 mg/ml. The dye solution was then incubated with TauO in a 1:2 ratio (w/w). The mixture was rotated overnight at 4 °C. The following day, the solution was centrifuged at 15,000 × *g* for 30 min using 10-kDa Amicon Ultra-0.5 centrifugal filter units to remove unbound dye. BDTOs were subsequently washed with PBS. The filter compartment was centrifuged to collect the concentrate.

### Seeding assay

Recombinant Tau 4R monomers were obtained by dissolving lyophilized pellets of recombinant 4R Tau at 1 mg/ml concentration in 1× PBS (86) and seeded with disease-relevant brain-derived tau oligomers from AD, DLB, or PSP brain tissues as described previously (51). The BDTO-TauM mixture was made at a ratio of 1:100 (w/w) with gentle agitation at room temperature for 2 days. After seeding, aliquots were taken and immediately used for Western blotting using T22 and Tau 13 antibody as well as AFM analysis for quality control. Total protein concentration was determined using a Pierce^TM^ BCA protein assay kit (Thermo Scientific, 23225) and stored at −20 °C until use.

### Preparation of BDTOs in the presence of curcumin derivatives

Disease-relevant tau oligomers were incubated with curcumin derivatives as described previously (48, 79, 80). A volume of 100 μl of BDTOs (1 μg/μl) was incubated with curcumin derivatives (final concentration 5 μm). Compounds were dissolved in EtOH 75%/DMSO (5:1) at a final concentration of 5 mm and diluted in 1× PBS or double-distilled H_2_O for incubation or toxicity assay (final concentration 5 μm). BDTOs in the presence of the small molecules and controls were incubated on an orbital shaker, without stirring, for 16 h under oligomerization conditions.

### Preparation of Aβ oligomers

AβO were prepared as described previously (24, 48). An amount of 0.3 mg of Aβ pellet was dissolved in 200 μl of hexafluoroisopropanol and incubated for 10–20 min at room temperature. The resulting solution was added to 700 μl of double-distilled H_2_O in a siliconized Eppendorf tube with holes placed on top of the cap to allow the slow evaporation of hexafluoroisopropanol. The samples were then stirred at 500 rpm using a Teflon-coated micro-stir bar for 48 h at room temperature in the fume hood.

### Filter trap assay

The filter trap assay was performed using a Bio-Dot SF microfiltration apparatus (Bio-Rad), as described previously (48, 80, 90). Briefly, 1 μg of each end-product reaction was applied onto nitrocellulose membranes, previously prewetted in TBS with very low Tween 0.01% (TBS-T), through the use of a vacuum-based bio-slot apparatus. Membranes were then blocked with 10% nonfat milk in TBS-T overnight at 4 °C. The next day, membranes were probed with the oligomer-specific tau antibody, T22 (1:250; in-house), misfolded tau aggregates T18 (1:5000; in-house), and sequence-specific tau antibodies Tau 13 (1:50,000; 835204, Biolegend), Tau 1 (1:5000; MAB3420, Millipore), Tau 46 (1:5000; 806601, Biolegend), HT7 (1:5000; Thermo Fisher Scientific, MN1000), Tau 5 (1:10,000; Biolegend, 806402), and anti-β-amyloid, 4G8 (1:1000; BioLegend, 800702), diluted in 5% nonfat milk for 1 h at room temperature. Membranes were then incubated with HRP-conjugated IgG anti-rabbit (1:10000) to detect T22, T18, and anti-mouse (1:10,000) secondary antibody to detect Tau 13, Tau 5, Tau 1, Tau 46, and HT7. Membranes were then washed three times in TBS-T, and ECL Plus (GE Healthcare) was used for signal detection.

### Indirect ELISA

An ELISA was conducted as described previously (24). Briefly, 96-well plates (Nunc Immobilizer, Amino Plates and Modules, 436006, Thermo Fisher Scientific) were previously coated with 1.5 μl of BDTOs in the presence or absence of curcumin derivatives using 50 μl of 1× PBS, pH 7.4, as coating buffer. After washing three times with TBS-T, plates were blocked for 2 h at room temperature with 120 μl of 10% nonfat milk in TBS-T. Plates were then washed three times with TBS-T and probed with 100 μl of primary antibodies for 1 h at room temperature (T22 (1:250) and T18 (1:2000)) and sequence-specific tau antibodies (Tau 13 (1:2000), Tau 5 (1:2000), Tau1 (1:2000), Tau 46 (1:2000), HT7 (1:2000), and 4G8 (1:1000)). Plates were then washed three times with TBS-T and incubated with 100 μl of HRP-conjugated anti-rabbit or anti-mouse IgG, diluted 1:10,000 in 5% nonfat milk in TBS-T, for 1 h at room temperature. Plates were washed three times with TBS-T and developed with 3,3,5,5-tetramethylbenzidine (S1599, Dako) The reaction was stopped using 100 μl of 1 m HCl, and absorbance was read at 450 nm using a POLARstar OMEGA plate reader. All experiments were performed in triplicate.

### Silver staining

1 μg of each BDTO was resolved on a precast NuPAGE 4–12% BisTris gel for SDS-PAGE (NP0335BOX, Invitrogen) and processed for silver staining (Pierce Silver Staining Kit, Thermo Fisher, 24612) for protein identification following the manufacturer's instructions.

### Western blotting

3 μg of each sample was resolved on a precast NuPAGE 4–12% BisTris gel for SDS-PAGE (NP0335BOX, Invitrogen) and transferred to nitrocellulose membranes. Then membranes were blocked with 10% nonfat milk in TBS-T overnight at 4 °C. After blocking, membranes were probed with sequence-specific tau antibody, Tau 5 (1:10,000; 806402, BioLegend), diluted in 5% nonfat milk for 1 h at RT. Membranes were then incubated with HRP-conjugated IgG anti-mouse (1:10,000, GE Healthcare) secondary antibody to detect Tau 5. ECL plus (GE Healthcare) was used for signal detection.

### PK digestion

Procedures for proteinase K digestion were described previously (49, 51, 56, 91). Briefly, in an Eppendorf tube, molecular grade water, Tris-HCl, and sodium chloride were added so that the final concentrations for these two buffers became 100 and 5 mm, respectively, in the entire solution volume. Next, BDTOs were added and treated with proteinase K enzyme at 0–1 μg/ml and incubated at 37 °C for 1 h. The enzymatic reaction was stopped by adding 1× LDS sample buffer (Invitrogen) followed by incubation at 95 °C for 5 min. Samples were then immediately transferred onto ice to stop the cleavage reaction. Samples were then ready to be loaded into 4–12% BisTris precast gel (Invitrogen) for SDS-PAGE or stored at −80 °C. Western blotting analysis with the sequence-specific Tau antibody, Tau 5, was performed to visualize the digested samples.

### Morphological analysis of BDTOs by AFM

BDTOs were characterized by AFM as described previously (24, 48, 56, 89). Briefly, samples were prepared by adding 5 μl of tau oligomers in the absence or presence of curcumin derivatives onto freshly cleaved mica and allowed to adsorb to the surface. Mica were then washed three times with deionized water to remove unbound protein and impurities and then air-dried. Samples were then imaged with a Multimode 8 AFM machine (Veeco, San Jose, CA, USA) using a noncontact tapping method (ScanAsyst-Air). AFM analyses were performed using the particle analysis tool of the NanoScope Analysis version 1.20rl AFM data processing software.

### Bis-ANS and ThT fluorescence

Fluorescence spectroscopy was performed as described previously (48, 80). Samples were prepared by adding 2 μl of BDTOs (0.3-0.5 μg/μl), alone or in the presence of CL3, and 248 μl of 10 μm bis-ANS (B153, Invitrogen), prepared in 100 mm glycine-NaOH buffer (pH 7.4), in a clear bottom 96-well black plate. Each experiment was performed in triplicate. The bis-ANS fluorescence intensity was measured at an emission wavelength of 520 nm upon excitation at 380 nm. For the ThT assay, samples were prepared using 2 μl of BDTOs (0.3–0.5 μg/μl), alone or in the presence of CL3, and 248 μl of 5 μm ThT (T3516, Sigma), dissolved in 50 mm glycine-NaOH buffer (pH 8.5). Each experiment was performed in triplicate. ThT fluorescence intensity was recorded at an emission wavelength of 490 nm upon excitation at 440 nm using a Polar Star Omega plate reader (BMG Labtech). Fluorescence spectra of the following solutions were measured as negative controls for both dyes (bis-ANS and ThT): dye alone, dye + vehicle. In addition, fluorescence spectra of dye + CL3 were measured to avoid any false positive readings due to the eventually intrinsic fluorescent properties of curcumin derivative. Each reading was corrected for the corresponding background fluorescence.

### Primary cortical neurons

Primary cortical neurons from C57BL/6 mice (Jackson Laboratory, stock number 000664) were maintained as described previously (48, 51, 91, 92). Briefly, cortical neurons were isolated from embryos at embryonic day 16–18 using Accutase solution (A6964, Sigma). Dissociated neurons were plated at a density of 3.0 × 10^4^ cells/well in 96-well plates containing high-glucose Dulbecco's modified Eagle's medium (10-013-CV, Corning) supplemented with 2% B-27 Plus (A3582801, Gibco), 10,000 units/ml penicillin, 10,000 μg/ml streptomycin, and 25 μg/ml amphotericin B (Gibco). After 2 h, plating medium was removed from cells and replaced with neurobasal medium (12348017, Gibco) plus 2% B27, 0.5 mm GlutaMax (35050-061, Gibco), 10,000 units/ml, 10,000 μg/ml streptomycin, and 25 μg/ml amphotericin B supplement. Cells were grown for 10–12 days *in vitro* before experiments, and 50% of medium changes were performed every 3 days. On day 10, neuronal cultures were ready for treatments.

## Cell toxicity assays

### MTS

Primary cortical neurons were grown on 96-well plates and exposed to increasing concentrations of BDTOs (0.125–1 μm) for 24 h to assess the toxic concentration for primary neurons.

Primary cortical neurons were then treated with 0.5 μm BDTOs or 0.5 μm BDTOs incubated with CL3 (final concentration 5 μm) and controls (TauM, vehicle, CL3) for 24 h.

The cytotoxic effect was determined using an MTS assay (G3582, Promega) for assessing cell viability following the manufacturer's instructions. Cell viability was corrected by the vehicle background. All measurements were performed in triplicate in three independent assays. Optical density (OD) was measured at 490 nm with a Polar Star Omega plate reader (BMG Labtech). Cell viability was calculated as the percentage of the OD value of treated cells compared with untreated controls, according to the equation, Viability = (OD SAMPLE/OD CONTROL) × 100%.

### LDH

Primary cortical neurons were grown on 96-well plates and treated for 24 h with 0.5 μm BDTOs or 0.5 μm BDTOs incubated with curcumin derivatives (final concentration 5 μm), and their cytotoxicity was assessed by evaluating LDH release using the Cytotoxicity Detection kit PLUS (Roche Applied Science, 04744926001). All measurements were performed in triplicate in three independent assays and corrected by the vehicle background. OD was measured at 490 nm with a Polar Star Omega plate reader (BMG Labtech). LDH release was calculated following the manufacturer's instructions.

### Immunofluorescence

Primary cortical neurons were plated at a density of 1 × 10^6^ cells/ml using poly-l-lysine–coated coverslips in 24-well plates as described previously (92). Cortical neurons were treated for 1 h with 0.5 μm BDTOs labeled with AF568 or 0.5 μm BDTOs labeled with AF568 pretreated with 5 μm CL3. After washing off unbound proteins, cells were fixed in 4% paraformaldehyde for 15 min at room temperature. Neurons were then washed three times with 1× PBS (10 min each) and permeabilized with 0.25% Triton X-100, diluted in 1× PBS for 10 min. After washing three times with 1× PBS, neurons were blocked with 5% goat serum for 1 h. Neurons were then immunostained overnight with the mature neuronal marker βIII-Tubulin (1:1000; ab78078, Abcam) at 4 °C. The next day, neurons were washed three times with 1× PBS and incubated with goat anti-mouse IgG AF488 (1:500, Thermo Fisher Scientific) for 1 h at room temperature. After washing with 1× PBS, neurons were mounted using Prolong Diamond Antifade mounting medium with DAPI (P36966, Invitrogen). Slides were then dried in the fume hood. Cortical neurons were imaged with a Keyence BZ-800 microscope using standard filters for DAPI, GFP, and Texas Red. A Nikon ×100 oil immersion objective was used to capture images.

For confocal microscopy, BDTO-treated cortical neurons were immunostained with βIII-Tubulin (mature neurons; *blue* in *merge*), and Rab5 (early endosome; *green* in *merge*). Images were captured with the Nikon ×63 oil immersion objective of a Zeiss LSM 880 confocal microscope using a 405-nm diode laser and argon laser 458/488/514 nm. High-resolution images were obtained with ZEN Black Lite software. Each treatment condition was randomly imaged in five different regions of interest and performed in duplicate.

### Cell fractionation

Primary neurons (5 × 10^5^ cells/ml) were cultured in a 6-well plate. After 24-h treatment with 0.25 μm oligomeric tau from DLB, AD, and PSP, alone or in the presence of CL3 (final concentration 5 μm), total cell lysates were collected using 1× radioimmune precipitation assay buffer (Cell Signaling, 9806) supplemented with 2% protease/phosphatase inhibitor (Roche Applied Science, 04906837001). Cells were then scraped and resuspended in ice-cold lysis buffer, subsequently centrifuged at 13,000 × *g* for 10 min for protein fractionation. The compartment extraction was conducted with a Qproteome cell compartment kit (Qiagen, 37502) as described previously (93). Cytoplasmic, plasma membrane, and nuclear proteins were isolated and quantified using Pierce^TM^ BCA protein assay kit (Thermo Scientific, 23225). Samples were loaded into 4–12% BisTris precast gels (Invitrogen) for SDS-PAGE. Western blotting analysis was performed by probing with generic tau antibody, total tau (ab64193, Abcam). β-Actin (A3854, Sigma), NMDAR1 (GTX133097, Gene Tex), and histone 3 (ab201456, Abcam) were used as loading and purity control of subcellular fractionation for the cytoplasmic, plasma membrane, and nuclear fractions, respectively.

### Tau RD P301S biosensor cell culture and treatment

Tau RD P301S biosensor cells (ATCC, CRL-3275) were cultured in Dulbecco's modified Eagle's medium supplemented with 10% FBS, 100 μg/ml penicillin, and 100 μg/ml streptomycin as described previously (51, 91). Cultures were maintained in a humidified atmosphere of 5% CO_2_ at 37 °C. For seeding assay, the cells were plated on poly-l-lysine–coated coverslips at a density of 1 × 10^5^ cells/well in a 24-well plate. After 24 h, cells were transduced with BDTOs/Lipofectamine 2000 (Invitrogen, 11668-027) or a BDTO + CL3/Lipofectamine 2000 mixture in Opti-MEM (Gibco, 31985-070) (75 nm BDTOs + 6 μl of Lipofectamine 2000 + Opti-MEM) for a total volume of 50 μl/well. The BDTO/liposome mixture was incubated at RT for 30 min before adding it to cells. Cells were incubated with transduction complexes for 24 h. After treatment, cells were washed multiple times with PBS, fixed with 4% formaldehyde for 15 min at RT, and mounted with Prolong Gold mounting medium. Slides were then dried in the fume hood. Cells were imaged with a Keyence BZ-800 microscope using standard filters for DAPI, GFP, and Texas Red. A Nikon ×40 objective was used to capture images.

### Statistical analysis

Western blotting signals were quantified using ImageJ. All data collected from Western blotting, filter trap assay, ELISA, and cell toxicity assays were subjected to statistical analyses and compared by one-way analysis of variance (ANOVA) followed by Dunnett's multiple-comparison test, unless otherwise specified, using GraphPad Prism 6.01 software. Results were considered statistically significant at *p* < 0.05. Each experiment was performed in triplicate (*n* = 3). All data collected from fluorescence assays (bis-ANS and thioflavin T spectroscopy binding assays) were subjected to statistical analyses and compared by one-way ANOVA followed by Dunnett's multiple-comparison test using GraphPad Prism 6.01 software.

## Data availability

All data are contained in the article.
